# Clinical findings and associated MRI findings of temporomandibular joint disc degeneration

**DOI:** 10.22514/jofph.2026.025

**Published:** 2026-03-12

**Authors:** Gürkan Ünsal, Ahmet Faruk Ertürk, Elif Meltem Aslan, Marco Di Blasio, Diana Russo, Gabriele Cervino, Maria Maddalena Marrapodi, Giuseppe Minervini

**Affiliations:** ^1^Division of Oral and Maxillofacial Radiology, Schulich School of Medicine and Dentistry, London, ON N6A 5C1 Canada; ^2^Department of Dentomaxillofacial Radiology, Biruni University, 34010 Istanbul, Türkiye; ^3^Department of Dentomaxillofacial Radiology, Lokman Hekim University, 06530 Ankara, Türkiye; ^4^Department of Biomedical, Surgical and Dental Sciences, University of Milan, 20122 Milan, Italy; ^5^Multidisciplinary Department of Medical-Surgical and Dental Specialities, University of Campania “Luigi Vanvitelli”, 80138 Naples, Italy; ^6^Department of Biomedical and Dental Sciences, Morphological and Functional Images, G. Martino Polyclinic, University of Messina, 98125 Messina, Italy; ^7^Department of Woman, Child and General and Specialized Surgery, University of Campania “Luigi Vanvitelli”, 80138 Naples, Italy

**Keywords:** MRI, Temporomandibular disorders, Disc degeneration

## Abstract

**Background**: Temporomandibular disorders (TMD) present with a wide spectrum of symptoms, and many of these are linked to structural changes within the temporomandibular joint (TMJ). Degenerative changes of the TMJ disc, including thinning, perforation, and positional changes, are common in symptomatic patients. MRI is the gold standard for evaluating soft tissues, but the relationship between clinical findings and specific MRI-detected disc degenerations remains unclear. This study aims to review the clinical symptoms reported by patients with TMD and evaluate if these symptoms are linked to MRI findings. **Methods**: Clinical examinations were conducted on patients presenting with TMJ discomfort between September 2019 and December 2023. Inclusion criteria were patients with suspected TMD who underwent bilateral TMJ MRI with both open- and closed-mouth scans. Exclusion criteria included history of head and neck radiotherapy, previous TMJ treatment or maxillofacial surgery, presence of intense artifacts, and lack of both open- and closed-position TMJ MRI scans. 180 TMJs from 90 patients (60 females, 30 males; mean age 33.5 years, range 15–59) were evaluated. Clinical symptoms such as pain, joint sounds, and trismus were recorded, and MRIs were assessed for TMJ disc position, disc displacement degree, disc degeneration, and TMJ effusion. **Results**: Statistically significant differences were found in trismus between sexes, with a higher incidence in females (*p* = 0.04). MRI revealed 112 joints with disc displacement with reduction (DDwR), 51 with displacement without reduction (DDwoR), and 17 healthy joints. Structural abnormalities were observed in 104 TMJs (57.8%), most frequently disc thinning. Pain and osteoarthritis were markedly more prevalent in DDwoR. **Conclusions**: This study confirmed significant links between clinical symptoms and TMJ disc degeneration. DDwoR was associated with more severe symptoms, including pain and osteoarthritis. MRI remains essential for diagnosing TMJ disorders and guiding treatment. Further prospective studies are needed to validate these findings.

## 1. Introduction

The temporomandibular joint (TMJ) is at risk for several disorders due to its complex structure, and temporomandibular disorders (TMD) involve conditions that affect both the TMJ and the masticatory muscles, leading to mechanical stress on the joint and triggering clinical symptoms [1, 2].

TMD can cause a wide range of clinical symptoms, including headaches, ear pain, and mood swings in addition to pain, trismus, and sounds during the jaw movements. These symptoms are often linked to degenerative alterations of the TMJ disc, including fissures, perforations, and other forms of structural damage. While magnetic resonance imaging (MRI) offers accurate vision of soft tissues conventional radiography has limited diagnostic utility for TMJ diseases. However, there are still very few studies looking at the complex relationships between different clinical symptoms and specific MRI-detected anomalies. Previous investigations primarily focused on pain perception, often overlooking correlations between various symptoms and detailed MRI-based evaluations of TMJ disc degenerations [1, 3].

Intra-articular positional irregularities and excessive stress on the TMJ can catalyze dysfunctional joint remodeling, initiating degenerative changes in the TMJ disc, a condition affecting a majority in mild forms but causing prolonged severe pain in some cases [1, 3].

Understanding these nuances is pivotal. This study aims to analyze multidimensional clinical symptoms in TMD patients, with MRI to assess and delineate correlations between a spectrum of symptoms and TMJ disc degenerations. This study aims to detect associations for enhancing TMD diagnosis and treatment approaches.

## 2. Materials and methods

### 2.1 Clinical examination

Ethical approval for this study was obtained from Lokman Hekim University under approval number 2024160 on 11 June 2024. Informed consent was obtained from all subjects and/or their legal guardian(s), and all data were de-identified before analysis.

Clinical examinations were performed on patients with suspected TMD who reported discomfort between September 2019 and December 2023. In addition to being assessed for pain, TMJ noises, and trismus, each patient filled out a survey describing the symptoms of their TMD.

Inclusion criteria were patients with suspected TMD who underwent bilateral TMJ MRI with both open- and closed-mouth scans.

Exclusion criteria were:

• The presence or history of head and neck radiotherapy.

• A history of TMJ treatment or maxillofacial surgery.

• MRI images with intense artifacts.

• Patients without open-position and closed-position TMJ MRI scans.

180 TMJs were obtained for assessment from a total of 90 patients (60 females and 30 males). The mean patient age was 33.5 years (range 15–59). During clinical examinations, orofacial pain was diagnosed as TMJ-related or masticatory muscle-related, following diagnostic criteria for TMD. Trismus was defined as unassisted mouth opening <35 mm with a history of limitation. TMJ sounds were classified as clicking or crepitus during jaw movement.

### 2.2 Magnetic resonance imaging

MRI was performed by utilizing a 1.5-Tesla scanner (Siemens Aera, Siemens Medical Systems, Erlangen, BY, Germany) with bilateral TMJ surface coils. In the sagittal plane, proton density-weighted turbo spin-echo images (PDWs) were captured with both closed- and open-mouth positions (TR (repetition time)/TE (echo time) 2770/35 ms), while T1-weighted turbo spin-echo images (T1Ws) were specifically obtained in the closed-mouth position (TR/TE 447/12 ms). In the axial plane, T2-weighted turbo spin-echo images (T2Ws) were acquired solely in the closed-mouth position (TR/TE 3520/68 ms).

#### 2.2.1 Disc displacement

Disc displacement was classified as:

• Healthy.

• Disc displacement with reduction (DDwR).

• Disc displacement without reduction (DDwoR).

#### 2.2.2 Degree of disc displacement

To evaluate the degree of disc displacement, measurements were taken with the jaw in a closed position. A line was drawn between the highest points of the back of the jaw joint and the raised part of the joint’s structure. The midpoint of the jaw joint was identified as the central point. Another line was drawn from this midpoint to the top-most part of the jaw joint, creating a vertical line. Also, a line was drawn from the back edge of the joint’s disc to its midpoint, referred to as the posterior line. The angle formed between this posterior line and a vertical line was used to measure the extent of disc displacement.

Degree of disc displacement was classified in closed-mouth position as:

• 0°–10°: Normal disc position.

• 11°–50°: Mild disc displacement.

• ≥51°: Severe disc displacement.

#### 2.2.3 Joint effusion

Joint effusion was classified in T2W fat-saturated images as:

• Absence of joint effusion.

• Presence of joint effusion.

#### 2.2.4 Presence of disc degeneration

Disc degeneration was classified as:

• Thinning.

• Disc Perforation.

• Disc Fracture.

• Absence of disc.

#### 2.2.5 Osteoarthritis

Osteoarthritis was classified as:

• Findings that suggest the presence of osteoarthritis.

• Findings that suggest the absence of osteoarthritis.

### 2.3 Clinical findings

Pain, trismus, and TMJ sounds were recorded as clinical findings, and MR images were interpreted in terms of disc position, degree of displacement, disc degeneration, and joint effusion. A blinded radiologist evaluated all MRIs.

### 2.4 Statistical analysis

SPSS version 21.0 (Statistical Package for Social Sciences, IBM Inc., Armonk, NY, USA) software was used for statistical analysis [4]. Chi-square test was used for evaluating differences between groups for categorical variables. A statistical significance level of *p * < 0.05 was adopted. All variables were categorical, and expected cell counts met chi-square test assumptions.

## 3. Results

180 TMJs from 90 patients (60 females, 30 males) were evaluated. Based on MRI findings, 112 joints (62.2%) exhibited disc displacement with reduction (DDwR), 51 (28.3%) showed displacement without reduction (DDwoR), and 17 (9.4%) were classified as healthy (Table 1). Structural abnormalities were detected in 104 TMJs (57.8%), most commonly disc thinning. Trismus was significantly more frequent in females (23/60, 38.3%) than males (7/30, 23.3%) (*p* = 0.04, Table 2).

**Table 1.** Examination of TMDs.

Derangement		DDwR		DDwoR		Healthy		p*
Extent								
	Normal	0	0.0%	0	0.0%	17	100%	<0.001
	Mild	94	83.9%	17	33.3%	0	0.0%	
	Severe	18	16.1%	34	66.7%	0	0.0%	
Structural Changes in TMJ Disc								
	Yes	62	55.4%	42	82.4%	0	0.0%	<0.001
	No	50	44.6%	9	17.6%	17	100%	
Types of Structural Changes in the TMJ Disc								
	Perforation	0	0.0%	15	29.4%	0	0.0%	<0.001
	Thinning	63	56.3%	26	51.0%	0	0.0%	
	NA	49	43.8%	9	17.6%	17	100%	
	Fracture	0	0.0%	1	2.0%	0	0.0%	
Pain								
	Yes	28	25.0%	41	80.4%	0	0.0%	<0.001
	No	84	75.0%	10	19.6%	17	100%	
Sound								
	Yes	98	87.5%	6	11.8%	0	0.0%	<0.001
	No	14	12.5%	45	88.2%	17	100%	
Bony Changes								
	Yes	30	26.8%	38	74.5%	1	5.9%	<0.001
	No	82	73.2%	13	25.5%	16	94.1%	
Effusion								
	Yes	6	5.4%	7	13.7%	0	0.0%	0.070
	No	106	94.6%	44	86.3%	17	100%	

*Chi-Square Test. p < 0.05. DDwR: Disc displacement with reduction; DDwoR: Disc displacement without reduction; TMJ: temporomandibular joint; NA: Not Available.

**Table 2.** Comparison of sexes in terms of trismus.

Sex		Male (n, %)	Female (n, %)	p*
Trismus				
	Yes	7, 23.3%	23, 38.3%	0.04
	No	23, 76.7%	37, 61.7%	

*Chi-Square Test, p < 0.05.

A comprehensive analysis of 180 TMJs was conducted, incorporating clinical and radiographic assessments, the severity of derangement, structural anomalies identified on MRI, presence of osteoarthritis, and effusion. Statistically significant findings were noted in all parameters except effusion (*p* = 0.07). Upon examining the data outlined in Table 2, it is seen that mild disc displacement was prevalent in joints presenting both DDwR and DDwoR.

Both DDwR and DDwoR cases often demonstrated structural abnormalities. Although perforation was infrequent, thinning of the disc was notably prevalent in both DDwR and DDwoR. Furthermore, a single case of disc fracture was identified. While pain was infrequently reported in DDwR, a higher incidence of pain was associated with DDwoR. Moreover, joints with DDwoR showed an increased occurrence of clicking sounds compared to those with DDwR. Additionally, the presence of bone surface degradation, such as osteoarthritis, was remarkably higher in DDwoR.

Upon contrasting clinical findings based on the presence of structural abnormalities identified in MRI statistically significant results were obtained in all parameters except for clicking sound (*p * < 0.05). Further analysis of the data provided in Table 3 revealed a notable prevalence of DDwR with structural abnormalities.

**Table 3.** Analysis of structural abnormalities in TMJ in terms of clinical findings.

Structure		Yes		No		p*
Derangement						
	DDwR	62	59.6%	50	65.8%	<0.001
	DDwoR	42	40.4%	9	11.8%	
	Healthy	0	0.0%	17	22.4%	
Extent						
	Normal	0	0.0%	17	22.4%	<0.001
	Mild	66	63.5%	45	59.2%	
	Severe	38	36.5%	14	18.4%	
Types of Structural Changes in the TMJ Disc						
	Perforation	15	14.4%	0	0.0%	<0.001
	Thinning	88	84.6%	1	1.3%	
	NA	0	0.0%	75	98.7%	
	Fracture	1	1.0%	0	0.0%	
Pain						
	Yes	52	50.0%	17	22.4%	<0.001
	No	52	50.0%	59	77.6%	
Sound						
	Yes	61	58.7%	43	56.6%	0.780
	No	43	41.3%	33	43.4%	
Bony Changes						
	Yes	54	51.9%	15	19.7%	<0.001
	No	50	48.1%	61	80.3%	
Effusion						
	Yes	3	2.9%	10	13.2%	<0.001
	No	101	97.1%	66	86.8%	

*Chi-Square Test. p < 0.05. DDwR: Disc displacement with reduction; DDwoR: Disc displacement without reduction; TMJ: temporomandibular joint; NA: Not Available.

Thinning of the disc was observed more frequently than perforation in joints with structural abnormalities. Pain accompanied structural abnormalities in half of the affected joints, with the incidence of bone surface degradation notably high in such cases.

Statistically significant results were obtained in all parameters when comparing other clinical findings with joints experiencing pain (*p * < 0.05). Upon examining the data outlined in Table 4, it was observed that DDwoR was particularly prevalent in joints experiencing pain.

**Table 4.** Analysis of clinical findings in joints with pain.

Pain		Yes		No		p*
Derangement						
	DDwR	28	40.6%	84	75.7%	<0.001
	DDwoR	41	59.4%	10	9.0%	
	Healthy	0	0.0%	17	15.3%	
Extent						
	Healthy	0	0.0%	17	15.3%	<0.001
	Mild	33	47.8%	78	70.3%	
	Severe	36	52.2%	16	14.4%	
Structural Changes in TMJ Disc						
	Yes	52	75.4%	52	46.8%	<0.001
	No	17	24.6%	59	53.2%	
Types of Structural Changes in the TMJ Disc						
	Perforation	15	21.7%	0	0.0%	<0.001
	Thinning	36	52.2%	53	47.7%	
	NA	17	24.6%	58	52.3%	
	Fracture	1	1.4%	0	0.0%	
Sound						
	Yes	24	34.8%	80	72.1%	<0.001
	No	45	65.2%	31	27.9%	
Bony Changes						
	Yes	41	59.4%	28	25.2%	<0.001
	No	28	40.6%	83	74.8%	
Effusion						
	Yes	11	15.9%	2	1.8%	<0.001
	No	58	84.1%	109	98.2%	

*Chi-Square Test. p < 0.05. DDwR: Disc displacement with reduction; DDwoR: Disc displacement without reduction; TMJ: temporomandibular joint; NA: Not Available.

Moreover, disorders in joints presenting pain were predominantly in advanced stages. Structural abnormalities were common in these cases. Among painful joints, disc thinning appeared more often than perforation. Bone surface damage was also frequently seen in painful joints, while clicking and effusion were relatively uncommon.

## 4. Discussion

Alterations in masticatory muscles, joint-disc integrity or both, can disrupt the complex structure of the TMJ and produce clinical and radiologic findings. Clinical manifestations in the TMJ make meaningful findings with MRI. It allows the determination of the position and morphology of the articular disc in the anteroposterior and mediolateral direction without ionizing radiation and the ability to visualize soft tissues with excellent resolution. It is stated that bone tissue can be adequately visualized through changes in signal intensities [1, 5, 6].

Joint-related symptoms (*e.g.*, clicking, pain) and masticatory muscle problems (*e.g.*, muscle discomfort, trismus) are among the many findings that patients with TMD can suffer from [7, 8, 9, 10]. Even though there are many studies in the literature examining the relationship between some of the clinical symptoms and changes in TMJ, based on existing evidence, there is no article examining the relationship between all clinical symptoms and TMJ degeneration [1, 3, 5, 7, 8, 9, 11]. Our study is the first of novelty in that it analyzed the relationship between a series of clinical symptoms and disc degenerations in the TMJ. In the current study, it was identified that DDwoR had more pain, clicks, osteoarthritis; mild form of disc displacement and disc thinning were more widespread in both DDwR and DDwoR.

Pain is the predominant TMD symptom and the most common reason for patients to consult the clinic [2, 12]. Emshoff *et al*. [13, 14] identified a correlation between pain and disc displacement, noting that the incidence of pain was higher in cases of DDwoR compared to DDwR; additionally, increased rates of osteoarthritis, edema and effusion were reported in DDwoR and the likelihood of pain was significantly increased in the presence of these. Jung *et al*. [15] determined that the probability of pain increased suggestively when the degree of ADD was ≥51°. Oğütcen-Toller *et al*. [16] have reported that degenerative condylar change increases with the degree of ADD and DDwoR was present in all cases. The disc is reversibly or irreversibly displaced anterior to the condyle in DDwR and DDwoR, with the posterior ligaments experiencing greater extension and tension compared to those in a normal disc position.

The ADD results in an overload on the retrodiscal tissue, which leads to increased free radical and nitric production in this area, resulting in amplified degeneration and inflammation of TMJ [11, 17, 18]. Therefore, because of this damage to the posterior ligament, the higher incidence of pain in DDwoR compared to DDwR in this study, in line with the literature, may be attributed to this reasoning.

The degree of disc degeneration and displacement is an essential indicator for potential TMJ disorder. Oğütcen-Toller *et al*. [16] concluded that degenerative condylar changes increased with the degree of disc displacement and intracapsular effusion was more often related with TMJ with DDwR. Roh *et al*. [19] noted a higher occurrence of degenerative changes and joint effusions in joints with DDwR or DDwoR compared to healthy TMJ. The present study revealed that disc thinning, and mild displacement were more common in joints with structural abnormalities.

Several studies in the literature have reported that osteoarthritis is not observed in patients without structural damage and that there is a high association between osteoarthritis and damage and that pain is dramatically increased in cases with osteoarthritis through structural damage [13, 15, 19, 20, 21, 22]. Campos *et al*. [21] demonstrated that osteoarthritis is not invariably a major aspect of TMJ pain, as degenerative bone changes can be seen also in asymptomatic TMJ. A multifactorial study by Emshoff *et al*. [13] showed that the impact of osteoarthritis on joint pain was not significant. Farina *et al*. [23] attributed this to the tendency of TMJ pain to diminish in advanced stages, as osteoarthritis primarily occurs at this stage. Our study found that osteoarthritis was more prevalent in DDwoR, and the incidence of pain was proportionally higher in these cases.

TMJ sounds are often the consequence of disturbance of the healthy condyle-disc relationship, though their clinical significance is not clear yet [3, 19]. Yasan *et al*. [6] detected clicking sounds in 13 of 36 patient with healthy disc position. These conclusions are in accordance with earlier studies showing that the occurrence of clicking in the TMJ is not solely a finding of disc displacement, as it can occur in TMJs with healthy disc-condyle relationship as well as in TMJs with DDwR [6, 24]. Morphological changes in TMJ surfaces, temporary adhesions, and mediolateral thickening of the TMJ disc, often undetectable through imaging but observable in arthroscopic operations, open surgeries, or autopsy samples, have been identified as additional causes of TMJ sound. Hence, systematic association of joint sounds with joint structure was difficult [6, 19].

The higher rate of trismus among females may indicate a biological influence. Since TMJ tissues include estrogen receptors, hormonal changes, particularly variations in estrogen levels, are thought to impact TMJ. Understanding these mechanisms can guide more effective, sex-specific approaches to TMD treatment. For example, higher levels of estrogen and progesterone during certain phases of the menstrual cycle might offer protective effects against TMJ pain, while lower levels during perimenopause could exacerbate symptoms. Understanding these factors can help tailor more effective, sex-specific treatments for TMD [25, 26].

Because this was a retrospective study, certain biases may exist. Using previously collected data can introduce selection bias and limit control over variables. Additionally, the exclusion criteria, such as previous TMJ treatment or maxillofacial surgery, could have eliminated cases with severe manifestations, thereby skewing the results. Recall bias may also be a factor, as patients’ self-reported symptoms may not be accurately remembered or recorded. Addressing these biases involves acknowledging their presence and discussing how they might impact the study’s findings, emphasizing the need for prospective studies to validate these results.

Integrating MRI into the initial assessment for TMJ pain and dysfunction is supported by the correlation between MRI results and patient symptoms. Clinicians should be aware that DDwoR is associated with more severe symptoms, including higher pain levels and osteoarthritis, and thus may require more aggressive treatment strategies. Early monitoring and timely treatment of mild disc displacement or thinning may help prevent more severe TMD. The findings also show how important it is for radiologists, dentists, and physical therapists to work together to offer comprehensive and well-coordinated care.

Prospective, long-term methodologies should be used in future research to better understand how TMDs arise and react to therapy. Examining hormonal, genetic, and environmental factors may reveal ways to prevent TMD, while evaluating treatment methods such as physical therapy, medication, and minimally invasive surgery could clarify their effectiveness. Comparative studies involving larger, more diverse populations are also necessary to generalize the findings and develop standardized diagnostic and treatment protocols for TMD.

## 5. Conclusions

In the current study, we inspected the correlations between multiple clinical symptoms and TMJ disc degenerations through detailed description using MRI; and found that DDwoR had more pain, and osteoarthritis; mild disc slippage and disc thinning were more common in both DDwR and DDwoR. Based on these observations, recognizing the correlation between the severity of clinical symptoms and structural damage in the TMJ enhances the role of clinical symptoms in guiding and preventing treatment. However, to overcome the limitations of this study, more comprehensive, comparative, and prospective clinical research is planned.
